# Geminivirus-Mediated Delivery of Florigen Promotes Determinate Growth in Aerial Organs and Uncouples Flowering from Photoperiod in Cotton

**DOI:** 10.1371/journal.pone.0036746

**Published:** 2012-05-15

**Authors:** Roisin C. McGarry, Brian G. Ayre

**Affiliations:** Department of Biological Sciences, University of North Texas, Denton, Texas, United States of America; Ohio State University, United States of America

## Abstract

**Background:**

Plant architecture and the timing and distribution of reproductive structures are fundamental agronomic traits shaped by patterns of determinate and indeterminate growth. Florigen, encoded by *FLOWERING LOCUS T* (*FT*) in Arabidopsis and *SINGLE FLOWER TRUSS* (*SFT*) in tomato, acts as a general growth hormone, advancing determinate growth. Domestication of upland cotton (*Gossypium hirsutum*) converted it from a lanky photoperiodic perennial to a highly inbred, compact day-neutral plant that is managed as an annual row-crop. This dramatic change in plant architecture provides a unique opportunity to analyze the transition from perennial to annual growth.

**Methodology/Principal Findings:**

To explore these architectural changes, we addressed the role of day-length upon flowering in an ancestral, perennial accession and in a domesticated variety of cotton. Using a disarmed *Cotton leaf crumple virus* (CLCrV) as a transient expression system, we delivered *FT* to both cotton accessions. Ectopic expression of *FT* in ancestral cotton mimicked the effects of day-length, promoting photoperiod-independent flowering, precocious determinate architecture, and lanceolate leaf shape. Domesticated cotton infected with *FT* demonstrated more synchronized fruiting and enhanced “annualization”. Transient expression of *FT* also facilitated simple crosses between wild photoperiodic and domesticated day-neutral accessions, effectively demonstrating a mechanism to increase genetic diversity among cultivated lines of cotton. Virus was not detected in the F_1_ progeny, indicating that crosses made by this approach do not harbor recombinant DNA molecules.

**Conclusions:**

These findings extend our understanding of FT as a general growth hormone that regulates shoot architecture by advancing organ-specific and age-related determinate growth. Judicious manipulation of *FT* could benefit cotton architecture to improve crop management.

## Introduction

Plant architecture is fundamental to agricultural productivity and artificial selection of desired growth habits is prominent in the earliest domestication of exotics into crops, the yield enhancements of the “green revolution”, and in modern crop improvement. Shoot architecture is determined by the fate of the apical meristems being indeterminate or determinate, the strength of apical dominance, branching pattern of lateral growth, and the timing and placement of reproductive growth. Indeterminate shoot apical meristems retain a population of vegetative stem cells indefinitely with tissue and organ differentiation occurring below and on the flanks. Because of this single point of continued growth, shoots derived from indeterminate apical meristems are said to be monopodial. In sympodial stems, the cells of the apical meristem undergo terminal differentiation, commonly in a flower or inflorescence, and the uppermost axillary bud continues the basic body plan of the shoot to produce a linear array of reiterative sympodial units along the axis of growth.

Cotton (*Gossypium* spp.) is the world’s most important textile fiber and a significant oilseed crop with a worldwide economic impact estimated at $500 billion annually [1]. *Gossypium* species are native to the arid and semi-arid regions of tropics and subtropics of both the old and new worlds, and includes approximately 45 diploid and 5 allotetraploid species [2]. They are long-lived perennials with architectural variation ranging from trailing and herbaceous to 15 m trees [2]. Most, if not all, are day-length sensitive and undergo repeated annual cycles of vegetative growth in long-day seasons with reproductive development triggered by short-day photoperiods, but cooler air temperature and dry seasons also commonly promote flowering [3].

Domestication of the two allotetraploids that comprise the majority of world-wide cultivations, *G. hirsutum* (upland or American cotton) and *G. barbadense* (Pima or extra-long staple cotton), initiated independently at least 5000 years ago [2], and the two have similar architectures. The main-stem apical meristem is monopodial and remains vegetative for the life of the plant. At each node, there is a leaf with stipules and two axillary buds: one of these generally remains dormant while the other may grow to form either a vegetative or reproductive axillary branch [4]. Vegetative branches are monopodial and reiterate the main stem while reproductive branches are sympodial and are called fruiting branches [5]. On fruiting branches, the apical meristem of each sympodial unit produces an internode, node, leaf with stipules (called the subtending leaf) and two axillary buds. The apical meristem then converts to a determinate floral meristem to produce a flower and ultimately a boll, and one of the axillary-bud meristems continues growth to form the next sympodial unit [4], [5].

Varieties domesticated for temperate climates were bred for day-neutrality and are cultivated and harvested as annual row crops [5]. This management strategy is well suited to highly mechanized production practices but is at odds with the plant’s inherent perennial nature. For example, vegetative growth continues after reproductive development initiates, and flower and fruit set are not synchronous but continue through the growing season. These competing sinks divert resources from fiber and seed production such that late-forming fiber is inferior to early-forming fiber and can discount crop value [5]–[7]. To control cotton’s perennial growth habit, growth inhibitors are used during the growing season and defoliants are used at season’s end to terminate the crop in preparation for mechanical harvest [6], [7].

In addition to retaining perennial growth habits, modern cultivated cotton suffers from restricted genetic diversity attributed to multiple bottlenecks during domestication and the current focus on a limited number of elite breeding lines [8]. Ancestral accessions, however, are a rich but underutilized source of variation affecting fiber quality and yield, and resistance to biotic and abiotic stresses [8]–[11]. Introgressing this diversity has potential for crop improvement but differences in the onset of flowering limit breeding to annual cycles in greenhouses or tropical territories unless photoperiod is artificially shortened. Some accessions require additional environmental cues, such as temperature, to initiate reproductive growth [3] and the specific conditions required may be difficult to replicate.

The photoperiodic pathway to flowering has many conserved elements irrespective of whether flowering is promoted by long or short days (reviewed by [12]–[15]). It is now well-established that the flowering hormone florigen, proposed by Mikhail Chailakhyan in the 1930s (see [16]) is the protein encoded by *FLOWERING LOCUS T* (*FT*) in Arabidopsis and its orthologs in other plants (e.g., *SINGLE FLOWER TRUSS* [*SFT*] in tomato and *HEADING DATE 3a* [*Hd3a*] in rice) [17]–[19]. Furthermore, *FT* orthologs govern the seasonal reproductive cycles of perennials [20], [21] and *FT* overexpression can overcome the extended juvenile stage of many flowering trees [22]–[25]. Florigen is not however only a flowering hormone but also promotes, in a dose-dependent fashion, a more determinate habit in all aerial organs including apical meristems, leaf primordia, and lateral meristems [26], [27]. Florigen is therefore a general growth hormone that contributes to the overall vegetative and reproductive architecture, and artificial selection at *FT* loci and related family members has contributed to domestication of several crops from exotic progenitors [21], [26], [28], [29].

Cotton presents a unique opportunity to analyze perennial and “annualized” plants from the same gene pool. With the long-term goal of characterizing the genetics of annual and perennial growth habits, we focused first on characterizing the conditions required for flowering in a perennial, ancestral, photoperiodic accession and in a domesticated, day-neutral variety of *G. hirsutum*. We demonstrate that day-length affects flowering, plant architecture, and leaf shape in a coordinated and reversible manner, leading us to hypothesize that these changes are florigen-dependent. To test the effects of florigen on cotton growth and to facilitate simple crosses between diverging lines, we developed a system for transient expression of the Arabidopsis *FT* gene from a disarmed *Cotton leaf crumple virus* (dCLCrV) vector in both domesticated and ancestral *G. hirsutum*. Ectopic expression of *FT* in both ancestral and domesticated cotton mimics the effects of day-length, suggesting that day-length functions mainly via FT in cotton. In addition, we show that “virus-induced flowering” (VIF) is a useful breeding tool to facilitate introgression of desirable traits from ancestral accessions into elite cotton cultivars. Together, our findings suggest that judicious manipulation of *FT* and related genes may enhance “annualization” and crop management by attenuating perennial characteristics.

## Materials and Methods

### 1. Plasmid Construction

Plasmid constructions were by standard procedures [30], [31] using *Escherichia coli* XL1-Blue (Stratagene, La Jolla, CA) as the host strain. Restriction endonucleases were from New England Biolabs (Beverly, MA), oligonucleotides were from Invitrogen (Carlsbad, CA), and *ExTaq* polymerase (TaKaRa, distributed by Fisher Scientific, Pittsburg, PA) was used for PCR amplification of plasmid components. DNA for cloning was purified with Wizard columns from Promega (Madison, WI). Clones incorporating a PCR product were sequenced to ensure accuracy (MWG, Huntsville, AL).

*FT* cDNA was reverse transcribed (SuperScript III, Invitrogen) and PCR amplified from Arabidopsis plants overexpressing *CONSTANS* [32] using oligonucleotides FT-fwd-*Spe*I (5′-ctcgtgactagtatgtctataaatataagagacc) and FT-rev nt559-*Nhe*I (5′-ctcgtggctagcaatatcaattggttataaagg), and sub-cloned in the *Spe*I and *Nhe*I sites (underlined) of pCLCrVJRT0008 (Genbank EU541443) [33] to construct an in-frame fusion with the start codon of the viral coat protein. Use of the *Spe*I restriction site resulted in the incorporation of Ala, Cys, Leu, Gln, Thr, Ser, and Met codons between Met-1 and the *FT* open reading frame. Plasmid DNA used for biolistic bombardment was prepared by standard alkaline lysis followed by column purification (Promega).

### 2. Plant Material and Growth Conditions

*G. hirsutum* Delta Pine 61 (DP61; GRIN PI 607174) and Texas 701 (TX701; GRIN PI 165329) seeds were a generous gift from Jack McCarty (USDA). To promote germination, seeds were delinted in concentrated sulfuric acid for 20 s, rinsed with running water for 10 min, neutralized with 100 mM sodium bicarbonate for 20 s, and rinsed with running water for 10 min. Delinted seeds were then heat-treated in an 80°C water bath for 1 min, plunged into ice water for 1 min, and allowed to air-dry before planting in MetroMix 366 potting medium (Sun Gro Horticultural, Bellevue, WA).

Biolistic bombardments used a PDS1000-He gene gun (Bio-Rad, Hercules, CA). 5 µg of each dCLCrV-A viral genome (dCLCrV::A, dCLCrV::αChl1 and dCLCrV::FT) was pre-mixed with 5 µg of CLCrV-B DNA, precipitated onto gold particles as per the manufacturer’s instructions (Bio-Rad), and stored overnight at −20°C. The abaxial surfaces of cotton seedling cotyledons aged 1–7 days post-germination (dpg), prior to emergence of the first true leaves, were bombarded using 900 psi and 1350 psi rupture disk pressures with the viruses adhered to 1.6 µm, 1.0 µm, and 0.6 µm gold particles. Four to six seedlings were bombarded with each treatment. 4 d after infection, bombarded and untreated seedlings were transferred to 4 gallon pots containing MetroMix 366 and grown under long-days (16 h light/8 h dark) in a greenhouse with supplemental light provided by a combination of metal halide and mercury lamps (up to 1300 µmol photons m^−2^ s^−1^ light intensity at leaf level) or short-day (10 h light/14 h dark) growth room conditions with metal halide, mercury, and T5 fluorescent lighting (200 µmol photons m^−2^ s^−1^ light intensity at leaf level). The efficiency of establishing an infection under each condition and with each vector is indicated in Fig. S1.

Cross-pollination using TX701 as the pollen donor and DP61 as the maternal line were conducted as suitably-aged flowers developed. DP61 flowers were emasculated at the “candle stage” (prior to anthesis when the petals remain tightly closed and extend past the sepals) [5], and the pistil was covered with a paper sleeve from a soda straw. Approximately 24 h later, the sleeve was removed and the pistils were pollinated using *FT*-induced flowers from TX701 plants. Self-pollination was allowed to occur in flowers not used for crosses. Seeds were removed from the mature bolls and were dried and stored at room temperature.

### 3. RNA and DNA Analyses

A leaf disk (100 mg) was removed from the unexpanded fifth true leaf of bombarded and untransfected cotton plants, homogenized with liquid nitrogen, and RNA extracted using the RNeasy Plant Mini Kit as per the manufacturer’s protocol (Qiagen, Valencia, CA). The RNA obtained was treated with RNase-free DNase (New England Biolabs) and cleaned with Trizol (Invitrogen) as per the manufacturers’ protocols. For first strand synthesis, 500 ng of RNA was used as template with a random hexamer primer and Superscript III reverse transcriptase as per the manufacturer’s protocol. One microliter of cDNA was used for PCR to detect *FT* and the internal reference gene *glyceraldehyde 3-phosphate dehydrogenase* A subunit (*GAPDH*) [33] with oligonucleotides FT-fwd nt51 (5′-gacgttcttgatccgtttaatag), FT-rev nt461 (5′-ccgagattgtagatctcagc), GAPDH fwd (5′-gggcaccatgactaccac), and GAPDH rev (5′-cagttgaagtcgggacg), for 25 cycles at 60°C annealing temperature using Phire DNA polymerase (Finnzyme, distributed by New England Biolabs).

A young leaf was removed from TX701, DP61, and F1 progeny plants, homogenized in liquid nitrogen, and genomic DNA extracted using CTAB followed with RNase A digestion [34]. PCR was performed using 0.5 µL of genomic DNA as template with oligonucleotides CLCrV-Rep-fwd (5′-taatcgccctcctcttggc), CLCrV-Rep-rev (5′-gacgccaacgccgtcaag), Chl-fwd (5′-cggtgacccttataactcgg), and Chl-rev (5′-gattacctgagccgatgag), and RedTaq Genomic DNA polymerase (Sigma, MO). CLCrV *Rep* and *Chl1* were amplified in the same 12.5 µL reaction for 30 cycles at 55°C annealing temperature.

## Results

### 1. Virus Induced Flowering Uncouples Determinate Growth from Photoperiod

**Figure 1:**
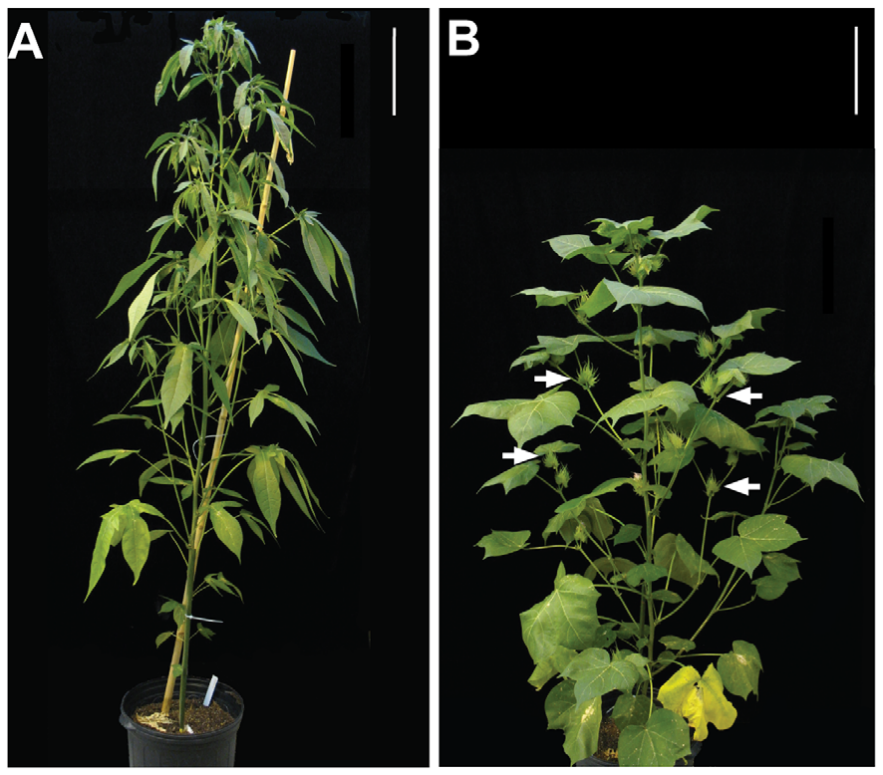
Wild and domesticated cotton have different architectures and flower at different times. (**A**) TX701, a photoperiodic accession, has deeply lobed “okra” leaves and pronounced apical dominance. TX701 does not flower under long days and this plant is completely vegetative**.** (**B**) DP61, a day-neutral cultivar, has a bushy growth habit with normal leaves and flowers profusely. Arrows are pointing to floral buds (“squares”). Both plants were grown under long-day conditions (16/8 h day/night) in a greenhouse with supplemental lighting. Scale bars, 25 cm.

Texas 701 (TX701) is a short-day *G. hirsutum* landrace that does not flower in the normal cotton growing season of the continental United States, but will produce fruit annually at the cotton winter nursery in Tecoman, Mexico [9]. In addition to being photoperiodic, TX701 has relatively long internodes and the monopodial vegetative branches grow close to vertical, giving plants a tall and columnar stature (Fig. 1A). Leaves are deeply lobed and are referred to as the okra-leaf shape. This is in striking contrast to the architecture of domesticated cotton, exemplified by DeltaPine 61 (DP61) used in this study. DP61 is day neutral and starts producing sympodial fruiting branches early in its life cycle. With the exception of the first, each sympodial unit (SU) of a fruiting branch grows from the axillary bud of the preceding SU. This gives fruiting branches a zig-zag appearance and contributes to greater horizontal spread relative to the vegetative branches that form only from the lowest buds. These features, along with large broad leaves and shorter internodes, contribute to a compact and bushy appearance (Fig. 1B).

Under short-day conditions in a growth room, TX701 plants developed fruiting branches from the main stem at node 20.6±3.1 (*n* = 7 plants; Table S1). This is the node of first fruiting branch (NFB), the first node above the cotyledons from which a fruiting branch develops, and this heritable measure reflects the “earliness” of the cultivar which is an important cropping characteristic [9]. Floral buds (*i.e.* squares) were first evident 92 days post-germination (dpg) and reached anthesis by 150 dpg. When grown under long–day conditions (16 hr light) in the greenhouse, TX701 did not flower by 146 dpg (when growth was terminated), but instead continued indeterminate growth to yield tall plants with numerous vegetative branches emerging from the main stem (Fig. 1A). In the same greenhouse conditions, DP61 NFB was at node 5.1±0.9 (*n* = 10) with squares evident at 33 dpg and anthesis at 64 dpg (Fig. 1B, Table S1).

**Figure 2:**
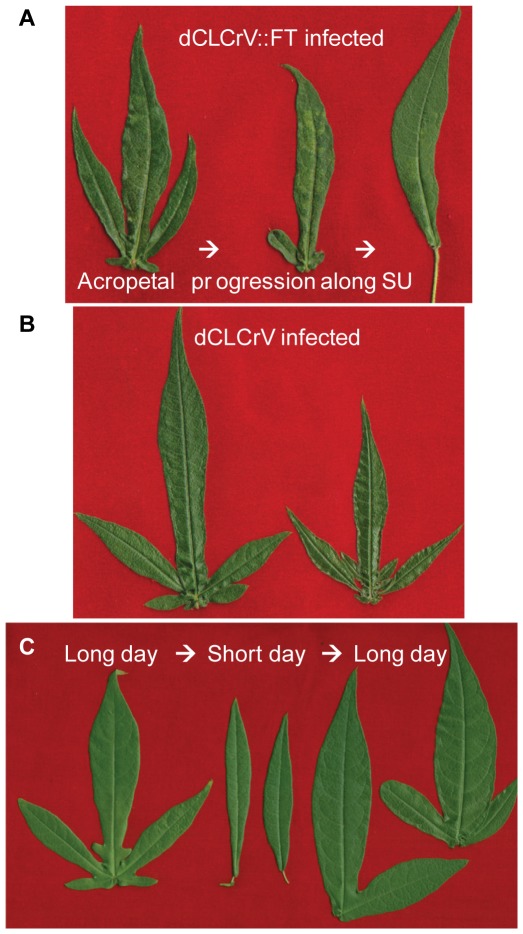
Florigen promotes determinate leaf growth. (**A**) Leaf shape along the fruiting branch (SU, sympodial units) of a dCLCrV::FT-infected TX701 plant transitions from highly lobed to lanceolate as the fruiting branch gets older. (**B**) dCLCrV-infected TX701 plants do not show the same transition. Note the leaf crumpling symptoms of dCLCrV-infected plants (**A**, **B**). (**C**) Changes in TX701 leaf correlate with changes in day length and reproductive *vs.* vegetative growth; these leaves are from the same plant that transitioned from long-day, to short day and back to long day growth conditions, with ∼10 weeks in each condition. See also Fig. S2 for plants grown exclusively in natural sunlight.

Day-length affected flowering time and leaf shape in a coordinated manner. When TX701 plants were moved from long-day to short-day conditions to induce flowering and bulk seed stocks, newly emerging leaves transitioned from the okra shape with deep palmate lobes to lanceolate simple leaves. Leaf shape transitioned back to deeply lobed when plants were returned to long day conditions (Fig. 2). These changes in leaf shape correlated with the photoperiodic onset, and subsequent termination, of reproductive growth. Development of lanceolate leaves also correlated with the onset of flowering on plants grown outside with only natural sunlight: lanceolate leaves and floral squares were evident by October 1, after the vernal equinox, and the transition was greater further along the fruiting branches (Fig. S2).

Given that the transition to reproductive growth was concurrent with altered leaf shape, we hypothesized that these changes were florigen-dependent and predicted that overexpressing Arabidopsis *FT* in ancestral, short-day cotton would uncouple flowering and determinate leaf growth from photoperiod. Generating transgenic cotton to ectopically express *FT* in ancestral lines is not practical since transformation is only efficient in domesticated Coker 312 (GRIN PI 529278) and regeneration takes about one year [35]–[37]. Rather, we utilized a disarmed *Cotton leaf crumple virus* (dCLCrV) vector to transiently deliver sequences of interest [33]. In this geminivirus-based system, the sequence of interest replaces the coat protein gene on the A genome and when co-delivered with the B genome, both replicate autonomously and spread systemically throughout the host as un-encapsidated DNA. The virus is considered disarmed since the coat protein is essential for whitefly-mediated plant-to-plant transmission. *AtFT* cDNA was cloned downstream of the coat protein promoter generating dCLCrV::FT, and, along with dCLCrV, an empty-vector control construct, and dCLCrV::αChl1 containing antisense sequence to the *G. hirsutum* magnesium chelatase subunit 1 [33], was used to infect TX701 seedlings.

**Figure 3:**
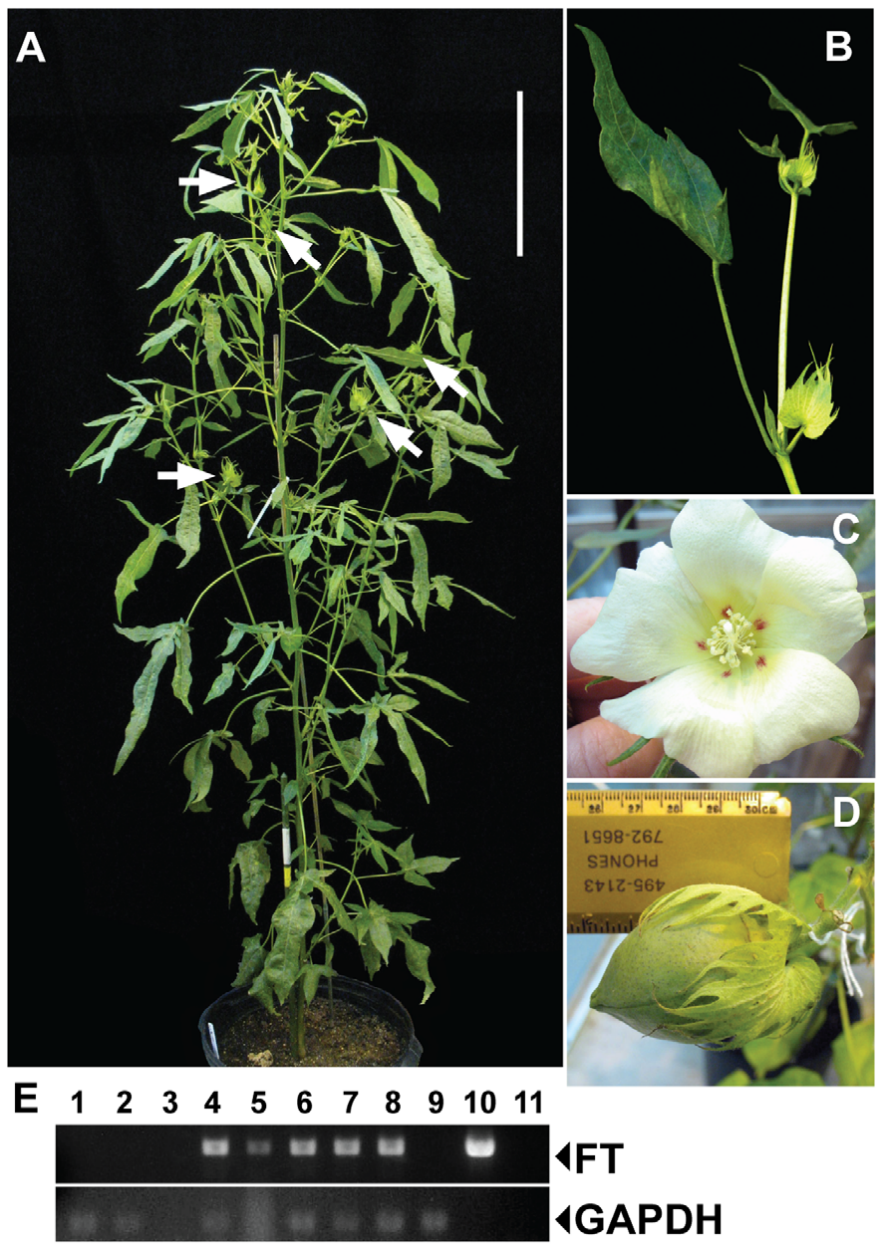
dCLCrV::FT infection uncouples flowering from photoperiod. (**A**) Arrows point to some of many squares on a representative dCLCrV::FT infected TX701 plant under long day conditions. Scale bar is 25 cm. (**B**) Close-up of sympodial growth on a fruiting branch of an FT-induced TX701 plant. (**C**) An open bloom from an FT-induced TX701 plant reveals characteristic dark red petal spots. (**D**) A boll on a DP61 plant resulting from cross-pollination with an FT-induced TX701 flower (See also Fig. S3). (**E**) RT-PCR demonstrates that *FT* expression is limited to plants inoculated with dCLCrV::FT; *GAPDH* expression serves as an internal control. Lanes are: 1 and 2, untransfected TX701; 3, no reverse transcriptase control; 4, dCLCrV::FT-infected plant shown in (C); 5, dCLCrV::FT-infected plant shown in (B); 6, dCLCrV::FT-infected plant shown in (A); 7 and 8, other dCLCrV::FT-infected TX701 plants that flowered (plants not shown); 9) dCLCrV::FT-bombarded TX701 plant which did not flower – note the absence of *FT*; 10) dCLCrV::FT plasmid template control; 11) no template control.

Under non-inductive, 16 hr-long days, TX701 plants infected with dCLCrV::FT were clearly reproductive and developed sympodial fruiting branches with productive flowers (Fig. 3, Fig. S1B, Table S1). Floral buds were evident by 33 dpg on node 5 of the main stem, but these earliest squares did not result in fertile flowers. The first flowers to reach anthesis did so at 71 dpg (Fig. 3C, Table S1). This timing compared favorably to DP61 plants which, under the same conditions, produced fruiting branches as early as node 5 and reached anthesis by 64 dpg. None of the untransfected TX701 plants, nor dCLCrV or dCLCrV::αChl1 infected TX701 flowered under these conditions. To correlate *FT* expression from dCLCrV::FT with flowering in TX701 plants, total RNA was isolated from the fifth leaf of the main stem at 29 dpg while it was an unexpanded sink leaf (*i.e.,* importing virus from infected mature leaves) and expression of *FT* determined by RT-PCR. *AtFT* expression was detected only in plants inoculated with dCLCrV::FT that subsequently flowered under non-inductive conditions (Fig. 3E).

Young VIF-treated TX701 plants had deeply lobed, palmate leaves on the main stem but more lanceolate leaves developed as the plants aged, and were more pronounced further along the fruiting branches (Fig. 2A). These changes in leaf shape were observed among 100% of flowering VIF-treated TX701 plants but not observed among dCLCrV-infected (Fig. 2B) and uninfected TX701 plants grown under long days, and imply that *FT* overexpression impacts leaf development. These observations that leaf marginal meristems in the okra-leaf background were suppressed by photoperiod (Fig. S2) and *FT* overexpression (Fig. 2) in an age-related fashion are consistent with models for plant development in which signals for determinate growth become stronger and/or more penetrant as the plant ages [27], [38]. Together, ectopic expression of *FT* in TX701 yields phenotypes similar to the pleiotropic effects of short day-length.

### 2. High Florigen Promotes an “Annualized” Cotton Phenotype

**Figure 4:**
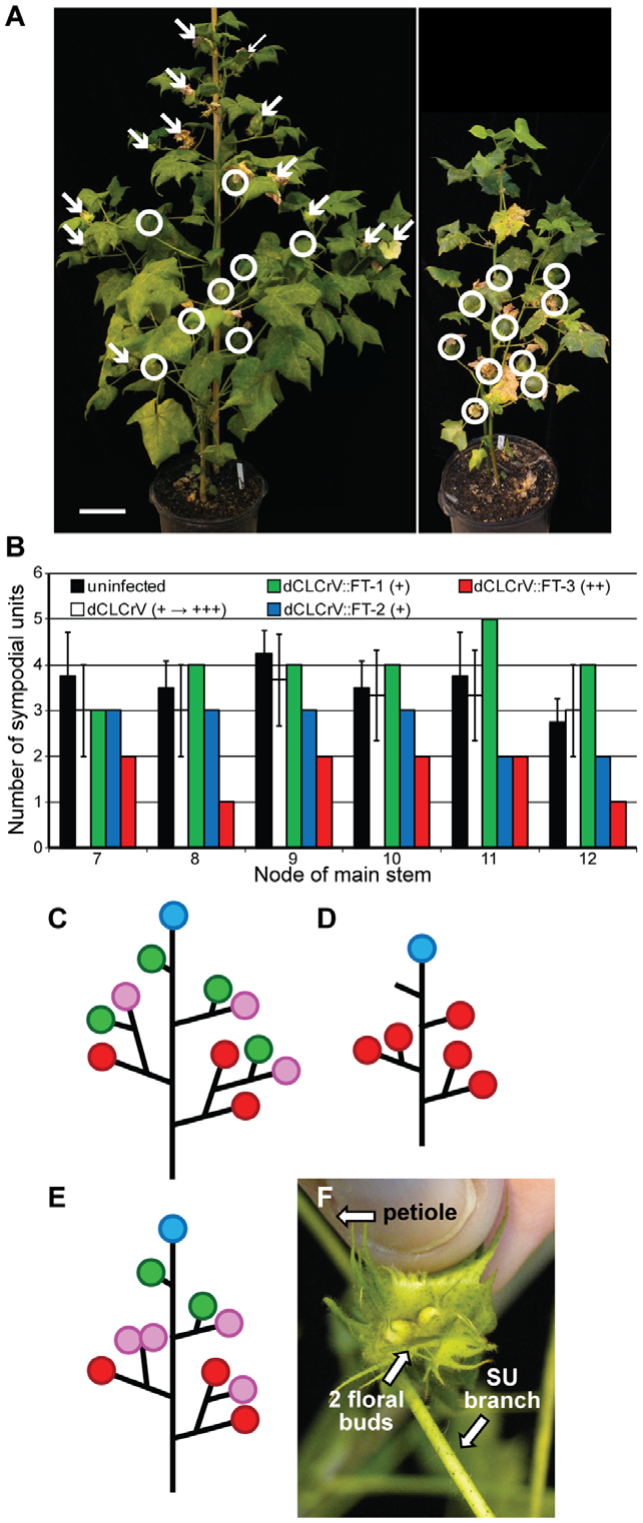
FT promotes determinate growth and synchronizes flowering. (**A**) dCLCrV::FT-infected DP61 plants exhibit a more compact growth habit. Shown are dCLCrV-infected (i.e., empty virus) and dCLCrV::FT-infected DP61 plants (left and right, respectively; both plants are 87 dpg and were grown at the same time in the same greenhouse). White circles highlight maturing bolls and arrows point to flowers before or in bloom. Note that the dCLCrV::FT-infected plant has only maturing bolls and no immature flowers. Scale bar is 25 cm. (**B**) dCLCrV::FT-infected plants demonstrate more determinate growth. Shown are the mean number of sympodial units along fruiting branches among untransfected plants (*n* = 4, black bar), dCLCrV-infected plants (*n* = 3, white bar), and three dCLCrV::FT-infected plants represented individually (green, blue and red bars) to show the range of variation; dCLCrV::FT-3 is the plant shown in (A). The severity of viral infection was scored as mild (+) or stronger (++) based on leaf crumpling. (**C, D, E**) Schematic representations of growth patterns observed among ∼90 d-old plants: **(C)** Uninfected and dCLCrV-infected DP61; (**D**) dCLCrV::FT infected DP61, (**E**) dCLCrV::FT infected TX701. Red circles represent maturing bolls; magenta circles represent immature or blooming flowers; green circles represent active buds reiterating sympodial growth; blue circles represent the monopodial bud of the main stem; and branches without a circle represent buds that have terminated without a flower or a fruit. Leaves are not represented, and the number of branches and internode lengths are not to scale. (**F**) Representative floral cluster (two floral buds inside a common bract whorl) terminating a fruiting branch on a dCLCrV::FT-infected TX701 plant. “SU branch” is the internode of the terminal sympodial unit; “petiole” is the petiole of the leaf subtending the floral cluster. No other vegetative growth is evident.

dCLCrV::FT infection made both wild and domesticated cotton more determinate in all aerial organs, consistent with observations in tomato overexpressing *SFT* [17], [27]. Relative to controls, DP61 plants infected with dCLCrV::FT had a more compact stature because internodes were shorter (data not shown) and fruiting branches had fewer sympodial units (Fig. 4A–D). These plants also had more synchronous flower and fruit set because fruit close to the main stem matured without continued formation of new flowers further along the fruiting branch (Fig. 4A–D). The extent of these phenotypes varied among infected plants, as expected given that dCLCrV does not infect every cell during systemic spread as evident from the chlorotic patches shown in a dCLCrV::αChl1-infected plant (Fig. S1A) and the severity of infection varies from plant to plant [33], [38]. For this reason, quantifying *FT* in individual buds was not pursued since levels in one bud do not predict levels in another, and destructive extractions preclude the possibility of monitoring bud fate. Rather, to measure growth in relation to dCLCrV::FT infection, whole-plant architecture phenotypes were correlated with the severity of leaf crumpling, which is a valid and commonly-used measure for virus titer [39] (Fig. 2 and Fig. 4B). The most compact plant architectures correlated with more robust dCLCrV::FT viral infection. A more determinate growth habit was not observed among plants infected with dCLCrV, despite leaf crumpling symptoms equal to or exceeding the severity of dCLCrV:FT infected plants, indicating that *FT* was indeed the cause of the growth alterations.

In the ancestral accession TX701, the fruiting branches of dCLCrV::FT-infected plants occasionally terminated with a floral cluster (Fig. 4E, F), which was not observed in short-day-induced plants (not shown) or in any DP61 plants. This is consistent with both the apical bud and one or both of the axillary buds of the terminal SU converting to a reproductive fate (*i.e.,* becoming determinate) roughly simultaneously before forming the vegetative components of the next SU (*i.e.,* internode and subtending leaf with new axillary buds). In both dCLCrV::FT-infected DP61 and TX701, the apical buds of the main stem and vegetative branches did not form flowers: the monopodial branches remained indeterminate (Fig. 4C–E).

As described above, NFB for TX701 was 20.6±3.1 (*n* = 7 plants) under short days and as early as 5 in long days with VIF (Table S1). This suggests that high florigen enhances earliness more effectively than photoperiod. Flowers and bolls also matured faster, but we attribute this to greater photosynthesis under long days rather than to dCLCrV::FT. NFB was also improved in day neutral DP61: 5.1±0.9 for untransfected plants (*n* = 10), 5.0±1.7 for dCLCrV-infected plants (*n* = 3), and 3.0±0.0 for dCLCrV::FT-infected plants (*n* = 3). Therefore, ectopic *FT* expression results in earlier NFB and more synchronized fruiting, both of which are important for cotton cultivation.

### 3. *FT*-induced TX701 Flowers can be Crossed with Day-neutral DP61 Flowers to Produce Fertile F1 Plants

**Figure 5:**
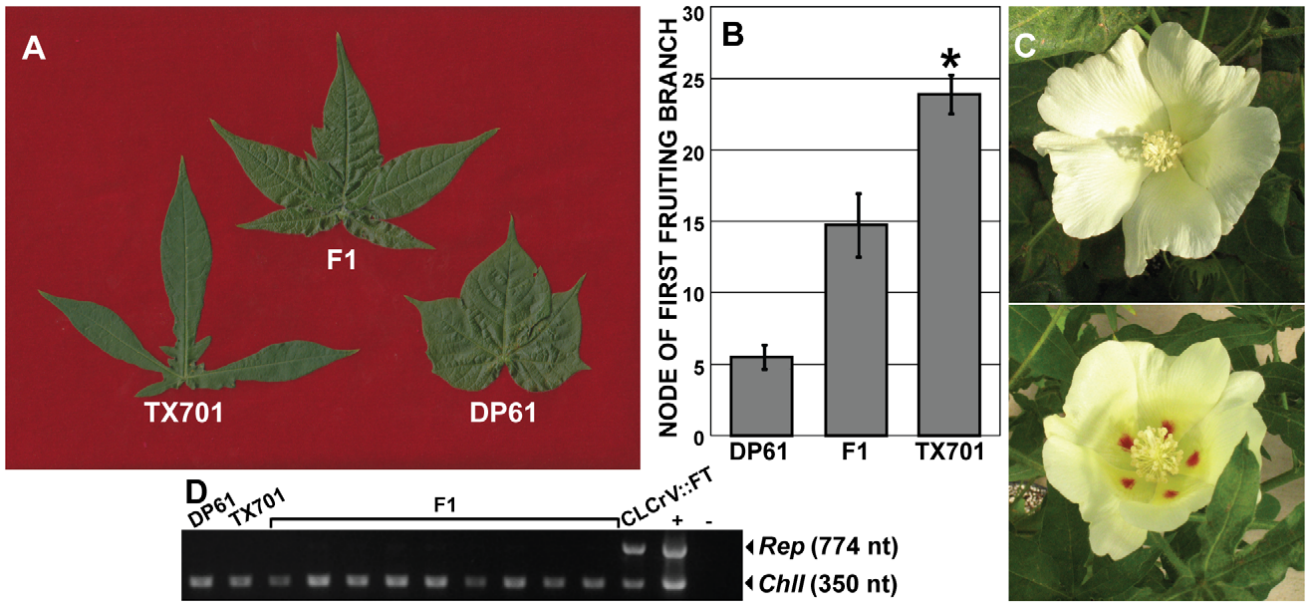
F_1_ progeny of crosses with a VIF-treated parent have expected traits and are virus-free. (**A**) The shape of main-stem leaves harvested from nodes 8–9 of TX701, DP61, and the F_1_, as labeled. TX701 leaves exhibit the okra leaf phenotype with five deep lobes and reduced lamina area. DP61 exhibits the normal leaf phenotype with three smaller lobes and a well-expanded lamina. The F_1_ from the DP61 × *FT*-induced TX701 cross exhibit an intermediate phenotype with leaves having five lobes of intermediate length. (**B**) Node of first fruiting branch is intermediate in the F_1_ population. Under non-inductive long days, the F_1_ produce floral buds (*n* = 46) in contrast to the TX701 parent which does not demonstrate reproductive growth by node 25 (*n* = 8 plants). Day-neutral DP61 produce floral buds earlier in development (*n* = 6 plants). (**C**) The maternal DP61 flowers (top) do not have the flower spots characteristic of the paternal TX701 pollen donor (see Fig. 3c); F_1_ progeny flowers (bottom) have the flower spot trait. (**D**) dCLCrV is not transmitted to the F_1_ progeny. From left to right, the viral *Rep* gene is not detected in uninfected DP61 or TX701 (lanes 1 and 2), nor in 9 different F1 progeny plants (lanes 3–11, collectively labeled F1), but is readily detected by PCR from a dCLCrV::FT-infected plant (lane 12, labeled dCLCrV::FT; this is the same plant shown in Fig. 3A and E, lane 6) and from a plasmid template (lane 13, labeled +). Detection of the endogenous magnesium chelatase subunit I (*ChlI*) serves as an internal control (plasmid control in the case of lane 13, +). No sequences were amplified in the absence of DNA (lane 14, labeled -).

Because flowering time was comparable between VIF-treated TX701 and DP61, we recognized that VIF could be used to introgress desirable germplasm into elite cultivated cotton lines. To test if VIF is a viable tool for breeding programs, we used *FT*-induced TX701 flowers as pollen donors in crosses with DP61. Cross-pollinated flowers formed healthy bolls with good seed yields (Fig. 3D and Fig. S3). To demonstrate that these crosses were successful, the F_1_ generation was scored for three traits: leaf shape, NFB, and petal spot. TX701 plants have an extreme okra-leaf phenotype with three to five deep and narrow lobes while DP61 plants have normal leaves with three broad lobes (Fig. 5A). The main-stem leaves from all DP61 × TX701 F_1_ plants (*n* = 46) exhibited an intermediate phenotype (Fig. 5A). Under 16 hr long-day conditions, NFB among DP61 plants was 5.1±0.9 (*n* = 10 plants), TX701 plants did not produce fruiting branches by node 25 when results were scored (*n* = 8 plants) and F_1_ NFB was 14.7±2.2 (*n* = 46; Fig. 5B), consistent with previous reports from conventional crosses between TX701 and DP61 [9]. TX701 flowers have creamy white petals with prominent burgundy petal spots (Fig. 3C, Fig. S3) while DP61 flowers are devoid of petal spots (Fig. 5C, top). All DP61 × TX701 F_1_ plants had prominent petal spots like the male parent (Fig. 5C, bottom). Each of these three phenotypes demonstrates that the F_1_ population was the result of the controlled crossing event between emasculated DP61 and *FT*-induced TX701 flowers.

In a previous study with plants infected with a dCLCrV::GFP construct, GFP fluorescence was sometimes observed in the outer boll wall, in the central column of the ovary, and in the ovule integuments [33]. Although it is well-established that geminiviruses are not ovule- or pollen-transmitted [40], [41], we screened the F_1_ generation by PCR for the presence of the viral *Rep* gene for confirmation. The dCLCrV *Rep* gene was absent from uninoculated DP61 and TX701 plants (Fig. 5D, lanes 1 and 2) and from all analyzed F_1_ plants, as expected (Fig. 5D, lanes 3 through 11). *Rep* sequences were detected exclusively in a TX701 plant infected with dCLCrV::FT and in a plasmid control (Fig. 5D, lane 12 and 13). Detection of the endogenous *Chl1*, present in all plant samples, served as an internal control (Fig. 5D). Consequently, although the pollen donor was manipulated with a recombinant virus, progeny of VIF plants do not carry DNA originating from the dCLCrV vectors, and should not be considered “genetically modified organisms” (GMOs).

## Discussion

FT is a key integrator of signaling pathways leading to floral induction. Extensive research demonstrates the conserved function of this gene product across diverse plant species irrespective of photoperiod. *FT* orthologs from a spectrum of eudicots have been expressed in Arabidopsis and induced early flowering, and *FT* orthologs expressed in species other than Arabidopsis have similarly promoted early flowering. For example, rice is a short-day monocot, but overexpressing *HEADING DATE 3a* in Arabidopsis under non-inductive conditions is as effective at promoting early flowering as overexpressing *FT* [42]–[44]. However, the function of FT is now considered in a broader context wherein it advances determinate growth in all organs. In this model, which was developed in perennial tomato [17], [27], local balances between *SFT* and *SELF-PRUNING* (*SP*), a potent *SFT*-dependent *SFT* inhibitor, control patterns of indeterminate and determinate growth: High *SFT/SP* expression ratios promote determinacy while low *SFT/SP* expression ratios promote indeterminacy. *SP* is a homolog of *Antirrhinum major CENTRORADIALIS* and Arabidopsis *TERMINAL FLOWER 1*. In this balance model, import of florigen (the product of *SFT*) into organs already expressing *SFT* communicates the status of distal tissues to modulate the *SFT/SP* ratio in new growth [17]. Importantly, high levels of florigen promote determinate growth in all organs and thus florigen functions as a general growth regulator rather than specifically a flowering hormone. In tomato, florigen confers different flowering responses in primary and secondary shoots, regulates the pattern of sympodial branches, accelerates the maturation and complexity of compound leaves, and promotes abscission zone formation, among other effects [27]. It is now apparent that the ratios of SFT and SP (FT and CEN/TFL1), as well as other members of the *CENTRORADIALIS/TERMINAL FLOWER 1/SELF-PRUNING* (*CETS*) gene family, have influenced the balance of vegetative (indeterminate) and reproductive (determinate) growth in many annuals and perennials, and have played important roles in the transition of wild exotics to domesticated crops [20], [21], [28], [29], [45].

Ectopic expression of *FT* affected growth in both photoperiodic TX701 and day-neutral DP61 cotton in ways that are consistent with the balance model for indeterminate and determinate growth regulation [17], [27], and thus contributes evidence that the balance model is broadly applicable. Using dCLCrV to deliver *FT* perturbed the existing balance to favor determinate growth, and the effects of high florigen included earlier flowering time uncoupled from photoperiod, the precocious transition to determinate plant architecture, and changes in leaf shape. Several TX701 fruiting branches terminated with a floral cluster rather than continuing sympodial reiterations (Fig. 4). We interpret this as roughly simultaneous conversion of the apical and axillary meristems to floral identity in the terminal sympodial unit, analogous to phenotypes observed in tomato with high SFT/SP ratios. Changes in leaf morphology, from deeply lobed palmate to simple lanceolate on the fruiting branches in response to both VIF and short-day induction, parallel the reduced complexity of compound leaves in tomato overexpressing *SFT* [27].

Flowers of VIF-treated TX701 frequently did not reach anthesis and many of those that did abscised before producing mature bolls (data not shown). Since *FT* promotes determinate growth and was expressed from a strong promoter on a multi-copy viral vector, floral maturation may have ceased because of premature termination. The accumulation of virus and *FT*-derived signal will vary from bud to bud as a function of viral replication and source/sink relationships, and we propose that those flowers that reached anthesis received a tolerable dose while those that terminated development received an excessive dose. It is worth noting that ectopic overexpression of the Arabidopsis *FT* in transgenic poplar [46] and from *Apple latent spherical virus* in apple [24] resulted in aberrant floral morphologies.

*FT* overexpression in DP61 resulted in fruiting branches developing at the third node, which was earlier than uninfected controls. However, since day-neutral cultivars already have NFB in the range of 5 to 7, this impact of increased FT levels on cotton agronomy will be modest. Of much greater significance is the role of *FT* overexpression on flower and fruit synchronization. Asynchronous flowering and fruit set throughout the growing season are indeterminate perennial characteristics that compromise yields and complicate crop management when cotton is grown as an annual row crop [5], [6], [47], [48]. *FT* overexpression reduced these perennial traits in DP61. Flowering and fruit maturation were more synchronized since the fruiting branches terminated rather than reiterating the pattern of sympodial units. This prevented continued production of new flowers while fruits closer to the main stem were maturing, and in combination with shorter internodes, resulted in more compact plants (Fig. 4). Unlike TX701, however, floral clusters were not observed at the termini of VIF-treated DP61 plants. The reason for this is not clear, but may relate to a higher balance of determinate growth factors in domesticated cultivars that result in cessation of bud growth before flower initiation. Flowers that did form on *FT*-overexpressing DP61 plants formed where expected (*i.e.*, at the ends of each sympodial unit) and unlike many of those on VIF-treated TX701, progressed through anthesis and formed mature bolls. This difference in DP61 and TX701 treated plants may also relate to different balances between determinacy and indeterminacy factors.

Our results presented here with cotton show that manipulating *FT* expression can alter growth habit in ways that would, with judicious control of expression levels, benefit yields and crop management: Earlier and more synchronized flowering and fruiting, a stockier growth habit, and a shorter growing season for focused agricultural inputs are all desired agronomic traits. In essence, achieving a more determinate and “annualized” growth habit is a prominent goal in modern cotton breeding. The fact that perennial traits of asynchronous flowering and continued vegetative growth remain, argues that, perhaps, traditional breeding has reached its limits, and that architectural manipulation through biotechnology will be required for further gains. Although yield per plant may be reduced, this could be compensated by denser planting, which in turn would have the benefits of faster field coverage to control weeds. Furthermore, the extent of vegetative growth and onset of reproductive growth could be customized to region-specific environments through manipulations of CETS. There already exists substantial support for these speculations. As noted, the CETS family is prominent in the domestication of several crops, and in tomato is directly linked to significant developments in the 20^th^ century. Tomato, like cotton, is naturally an indeterminate perennial with continued flower and fruit set through the growing season. However, the *sp* mutant of tomato, which results in a high SFT(FT)/SP(TFL) ratio, has accelerated termination of sympodial growth, and results in a more compact, determinate plant with nearly homogeneous fruit set. Identifying the *sp* phenotype [49] “was the single most important genetic trait in the development of modern agrotechniques for this crop plant because the ‘determinate’ growth habit facilitates mechanical harvest” [50], [51].

It was previously shown that expressing Arabidopsis *FT* from *Zucchini yellow mosaic virus* and *Apple latent spherical virus* promoted flowering under non-inductive photoperiods in melon [52] and soybean [53], respectively, and reduced the juvenile phase of apple trees [24]. In addition to promoting determinate cotton plant architecture, we and others [24] demonstrate that VIF is a promising breeding tool. Domestication of wild species often contributes to genetic homogeneity in crops, and loss of diversity is associated with loss of tolerance/resistance to abiotic and biotic stresses, and increased susceptibility to pathogens, pests and environmental change. The Irish potato famine, caused by the rapid spread of *Phytophthora infestans* among genetically-uniform cultivated potatoes [54] and over-exploitation of the Tcms cytoplasm in maize, which confers cytoplasmic male sterility but also susceptibility to *Helminthosporium maydis*, the causative agent of southern corn leaf blight [55], are classic examples of how reliance on inbred crops can have devastating agricultural and social impacts. Genetic diversity across the *Gossypium* genus has been estimated and there is consensus that domesticated lines are highly inbred while wild races contain extensive diversity [2], [10], [11], [56]. The two species of highest economic importance, *G. hirsutum* and *G. barbadense*, are both derived from a common allotetraploid and this polyploidization event was the first major bottleneck [2]. Moreover, Upland cotton, which accounts for ∼90% of USA production, derives from race ‘latifolium’ [10]. Spread of cotton boll weevil early in the twentieth century further limited diversity [10], and pedigrees of commercially successful modern lines indicate frequent use of relatively few parents [11], [56]. Furthermore, most transgenic cultivars contain a linkage group of the transgene donor parent, Coker 312 [56]. Based on this history, it is apparent that hybridization with lines outside of ‘latifolium’ will be required to increase diversity in Upland cotton. Since these are photoperiodic, VIF will benefit breeding efforts. For that same reason, VIF may also accelerate the generation of recombinant-inbred lines for linkage-association mapping and nested-association mapping [57]–[59], and reduce germplasm maintenance costs by permitting self-fertilization of photoperiodic or long-seasoned accessions in temperate regions.

In summary, the *FT* gene product, florigen, is increasingly recognized as a general growth hormone that regulates shoot architecture by advancing organ-specific and age-related determinate growth via its local expression and systemic distribution (as reviewed in [60]). It is now clear that the ratio of florigen to other members of the same gene family evolved to influence patterns of vegetative and reproductive growth in annual [28], biennial [21], and perennial plants [20], and that changes in these ratios contributed to the domestication of many crops from wild progenitors [28], [29]. Knowledge of these expanded roles beyond control of photoperiodic flowering opens new avenues for exploiting florigen with the applied goals of enhancing plant productivity and crop management. We demonstrate here that virus-induced flowering (VIF) promotes determinate growth: earlier and photoperiod-independent flowering, precocious transition to reproductive architecture, and lanceolate leaf shape were coordinated with ectopic expression of *FT* in ancestral cotton; and greater fruit synchronization and “annualized” growth were observed in domesticated cotton infected with dCLCrV::FT. Our results indicate that judicious manipulation of *FT* and related genes could benefit cotton architecture to improve crop management.

## Supporting Information

Figure S1 **Biolistic delivery of dCLCrV::αChl1 and dCLCrV::FT promotes silencing and flowering, respectively, in TX701.** (**A**) Cotton plants inoculated with dCLCrV::αChl1 show systemic and sustained silencing of the magnesium chelatase 1 (Chl1) subunit, resulting in chlorotic sectors. Shown are 6-week old TX701 plants that were inoculated with dCLCrV (left) and dCLCrV::αChl1 (right) at the seedling stage. (**B**) Biolistic rupture disk pressure and size of gold particles affect transfection of TX701 cotton. Shown is the percentage of plants exhibiting silencing when inoculated with dCLCrV::αChl1 adhered to different sized gold particles and delivered at 900 and 1350 psi (for each treatment, *n* = 4). Also shown is the percentage of TX701 plants that flowered under non-inductive long days when inoculated with dCLCrV::FT using the same biolistic parameters (*n* = 4 for each treatment except with 0.6 µm gold and 1350 psi where *n* = 6).
(TIF)

Figure S2 **Leaves from TX701 plants grown outside with only natural sunlight.** (**A**) Main stem leaf that formed under long day conditions while plants had no reproductive growth. (**B**) A fruiting branch from the same plant that developed under short day conditions. Note the floral squares and the simpler, more lanceolate subtending leaves towards the tip of the branch. Scale bars, 5 cm.
(TIF)

Figure S3 **Cross-pollinating emasculated DP61 flowers with** ***FT*** **-induced TX701 male parents.** (**A**) Flowers from dCLCrV::FT-infected TX701 plants were used as pollen donors to (**B**) cross-pollinate emasculated DP61 flowers. (**C, D**) Healthy bolls formed with good seed set (**E**).
(TIF)

Table S1 **VIF accelerates the time to flower irrespective of photoperiod in short-day TX701 and day-neutral DP61 cotton.**
(DOC)
