# Supplementary figures and images for: Geminivirus-Mediated Delivery of Florigen Promotes Determinate Growth in Aerial Organs and Uncouples Flowering from Photoperiod in Cotton
Source: PLoS One. 2012 May 15;7(5):e36746. doi: 10.1371/journal.pone.0036746 (PMC3352926; doi:10.1371/journal.pone.0036746)

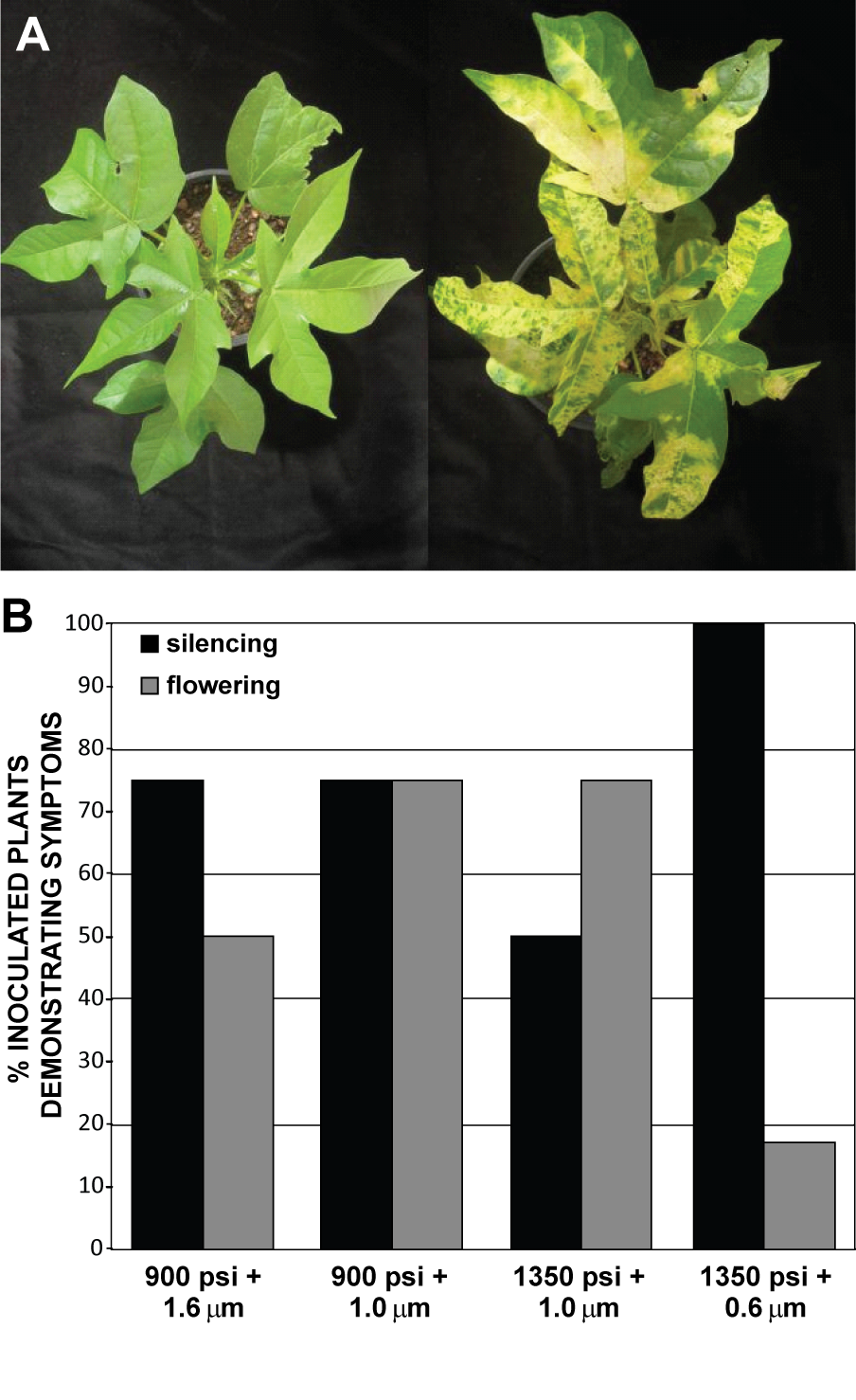

Supplement: Figure S1 — Biolistic delivery of dCLCrV::αChl1 and dCLCrV::FT promotes silencing and flowering, respectively, in TX701. (A) Cotton plants inoculated with dCLCrV::αChl1 show systemic and sustained silencing of the magnesium chelatase 1 (Chl1) subunit, resulting in chlorotic sectors. Shown are 6-week old TX701 plants that were inoculated with dCLCrV (left) and dCLCrV::αChl1 (right) at the seedling stage. (B) Biolistic rupture disk pressure and size of gold particles affect transfection of TX701 cotton. Shown is the percentage of plants exhibiting silencing when inoculated with dCLCrV::αChl1 adhered to different sized gold particles and delivered at 900 and 1350 psi (for each treatment, n = 4). Also shown is the percentage of TX701 plants that flowered under non-inductive long days when inoculated with dCLCrV::FT using the same biolistic parameters (n = 4 for each treatment except with 0.6 µm gold and 1350 psi where n = 6). (TIF) [file pone.0036746.s001.tif]

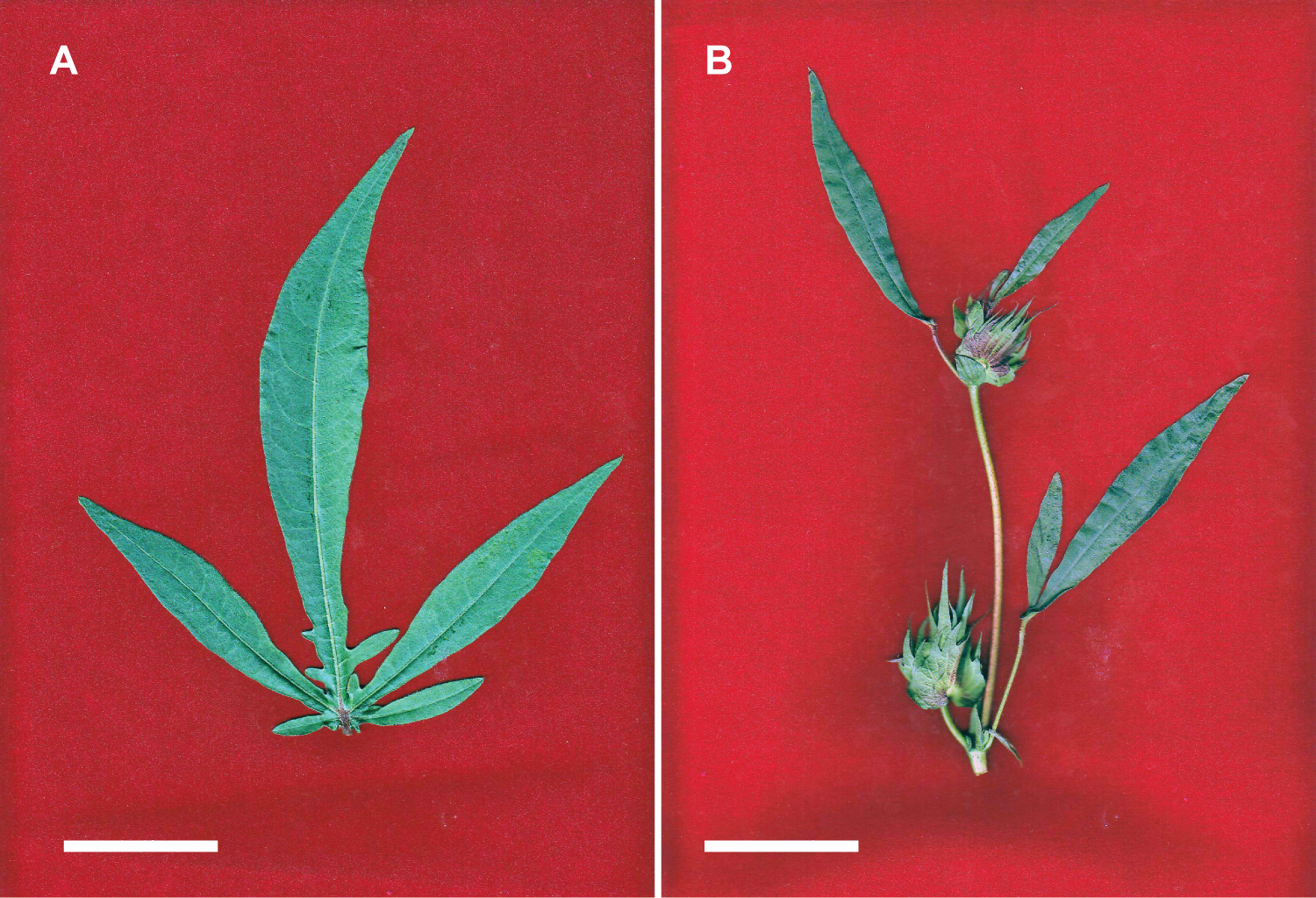

Supplement: Figure S2 — Leaves from TX701 plants grown outside with only natural sunlight. (A) Main stem leaf that formed under long day conditions while plants had no reproductive growth. (B) A fruiting branch from the same plant that developed under short day conditions. Note the floral squares and the simpler, more lanceolate subtending leaves towards the tip of the branch. Scale bars, 5 cm. (TIF) [file pone.0036746.s002.tif]

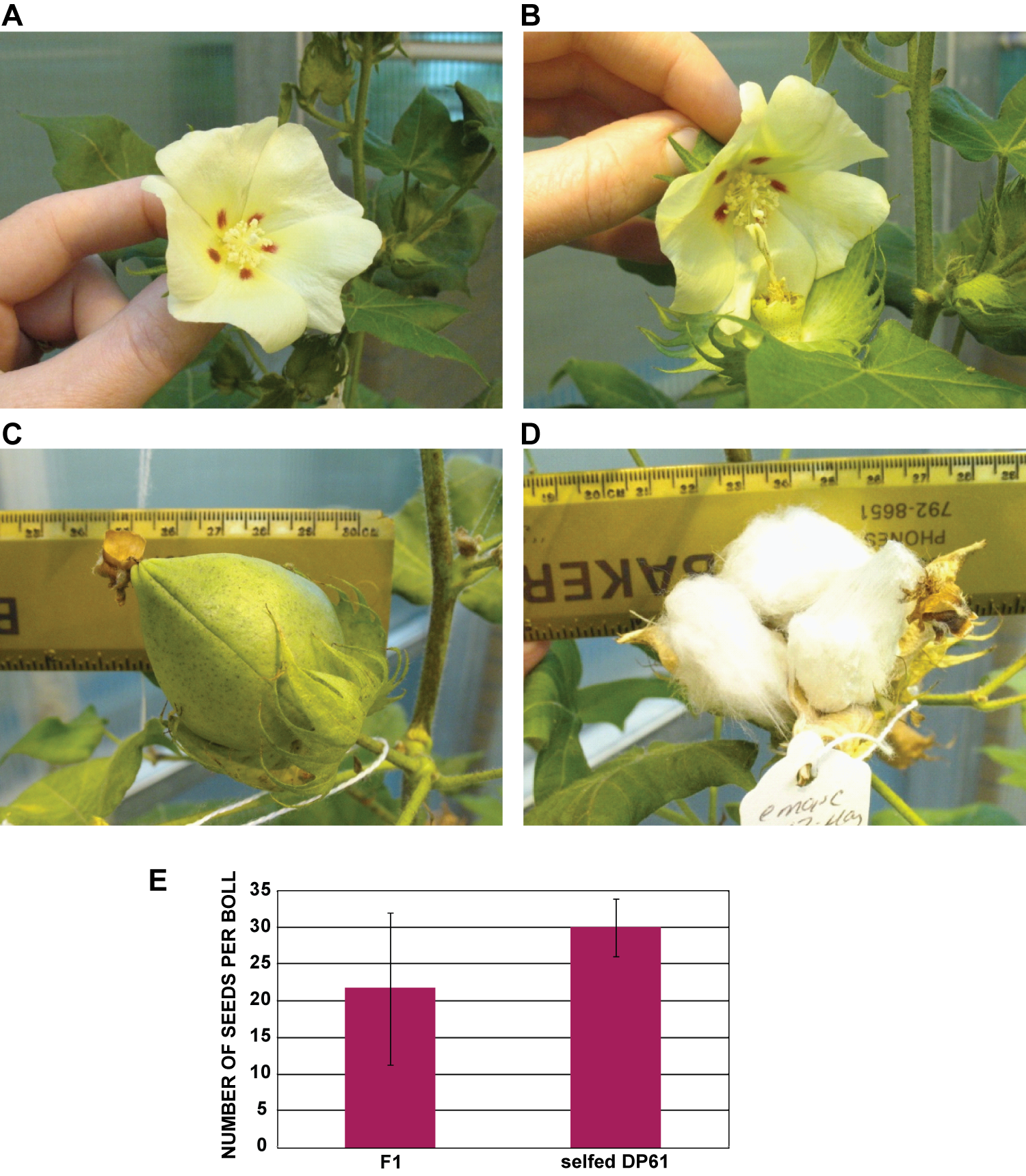

Supplement: Figure S3 — Cross-pollinating emasculated DP61 flowers with FT -induced TX701 male parents. (A) Flowers from dCLCrV::FT-infected TX701 plants were used as pollen donors to (B) cross-pollinate emasculated DP61 flowers. (C, D) Healthy bolls formed with good seed set (E). (TIF) [file pone.0036746.s003.tif]
