# Supplementary material for: Geminivirus-Mediated Delivery of Florigen Promotes Determinate Growth in Aerial Organs and Uncouples Flowering from Photoperiod in Cotton
Source: PLoS One. 2012 May 15;7(5):e36746. doi: 10.1371/journal.pone.0036746 (PMC3352926; doi:10.1371/journal.pone.0036746)
Supplement: Table S1 — VIF accelerates the time to flower irrespective of photoperiod in short-day TX701 and day-neutral DP61 cotton. (DOC) [file pone.0036746.s004.doc]

**Supplementary Table S1**

| **ACCESSION** | **FIRST FLORAL BUDS (dpg)** | **FIRST ANTHESIS (dpg)** | **NFB** |
| --- | --- | --- | --- |
| TX701- inductive  SD (10/14 h) | 92 | 150 | 20.6 ± 3.1 (*n* = 7) |
| TX701 –non-inductive  LD (16/8 h) | >146* | N/A | N/A |
| TX701 – VIF –  non-inductive LD (16/8 h) | 33 | 71 | 9.9 ± 4.8 (*n* = 9) |
| DP61 (16/8 h) | 33 | 64 | 5.1 ± 0.9 (*n* = 10) |
| DP61 – VIF (16/8 h) | nd | nd | 3.0 ± 0 (*n* = 3) |

* With non-inductive long days (16/8 h), TX701 was completely vegetative at 146 dpg.

SD = short days; LD = long days; N/A = not applicable; nd = not determined
